# Limiting Trial‐and‐Error: Linker‐Based 2D Descriptor Analysis to Support Very Early Proteolysis‐Targeting Chimeras Design

**DOI:** 10.1002/cmdc.70532

**Published:** 2026-10-11

**Authors:** Giulia Apprato, Serena Traversa, Giulia Caron, Giuseppe Ermondi

**Affiliations:** ^1^ Department of Molecular Biotechnology and Health Sciences University of Turin Torino Italy

**Keywords:** degradation, linker, molecular properties, PCA, PROTAC

## Abstract

Targeted protein degradation has emerged as a clinically validated therapeutic strategy, culminating in the recent FDA approval of the first proteolysis‐targeting chimeras (PROTAC) drug (VEPPANU). Within PROTACs, the linker plays a central role in modulating degradation efficiency as well as physicochemical and in vitro absorption, distribution, metabolism, and excretion properties, yet linker optimization still largely relies on empirical “linkerology” approaches. Here, we investigated whether simple 2D linker descriptors could provide informative insights for very early‐stage PROTAC design. Four matched PROTAC series, including both cereblon‐ and von Hippel–Lindau‐recruiting degraders, were retrospectively analyzed using a workflow combining the in‐house Bellerophon‐based linker extraction with principal component analysis. Selected descriptors captured linker hydrophobicity, polarity, flexibility, and topology and were correlated with experimental degradation, permeability, solubility, lipophilicity, protein binding, and metabolic stability data. In several datasets, linker‐dependent trends associated with the molecular properties of degraders and their degradation efficiency were identified, particularly for von Hippel–Lindau‐based PROTACs. Although the limited size of currently available datasets prevents broad generalization, this work highlights the potential of simple descriptor‐based analyses to support early candidate prioritization and reduce unnecessary synthetic efforts.

## Graphical abstract

Can simple linker descriptors guide PROTAC design? Analysis of matched CRBN‐ and VHL‐recruiting degraders shows that basic 2D linker properties may capture trends linked to degradation efficiency, in vitro ADME and physicochemical properties. A practical approach to reduce trial‐and‐error in early targeted protein degradation programs.

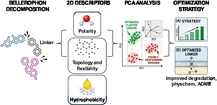

## 1 Introduction

Targeted protein degradation (TPD) has emerged as a powerful therapeutic strategy capable of modulating disease‐associated proteins through catalytic elimination rather than conventional occupancy‐driven inhibition [1]. Among TPD modalities, proteolysis‐targeting chimeras (PROTACs) have gained considerable attention because of their ability to recruit an E3 ubiquitin ligase, most commonly cereblon (CRBN) or the von Hippel–‍Lindau (VHL) E3 ligase complex, thereby promoting ubiquitination and subsequent proteasomal degradation of the protein of interest (POI) [2]. This mechanism offers several advantages over traditional small‐molecule inhibitors, including prolonged pharmacological effects, the potential to overcome resistance mutations, and the possibility to target proteins that are difficult to inhibit through classical approaches. The TPD field has rapidly evolved from a conceptual strategy into a clinically validated therapeutic modality. Importantly, in May 2026, the FDA approved Vepdegestrant (VEPPANU), the first PROTAC therapeutic for the treatment of ESR1‐mutated advanced breast cancer, representing a major milestone for the field and establishing the TPD modality as a clinically validated drug discovery strategy [3].

From a structural point of view, PROTACs are heterobifunctional molecules composed of a ligand for the POI (the warhead), an E3 ligase recruiter (E3 ligand), and a linker connecting the two moieties. Although extensive efforts have focused on optimizing the affinity and selectivity of the ligands, increasing evidence indicates that the linker is a critical determinant of degrader’s future as a drug [4]. First, linker length, composition, flexibility, and attachment vectors strongly influence the formation and stability of the ternary complex, ultimately affecting degradation and selectivity. For instance, studies from Ciulli and coworkers demonstrated that PROTAC‐induced protein–protein interactions and linker‐dependent conformational arrangements can strongly impact ternary complex cooperativity and selective degradation [5]. Second, the linker deeply affects the in vitro absorption, distribution, metabolism, and excretion (ADME) and physicochemical properties of PROTACs [6]. As an example, a recent work by Kihlberg and coworkers highlighted the importance of linker‐dependent conformational behavior in determining PROTAC permeability [7]. These observations suggest that linker optimization is a critical step to designing effective PROTAC drug candidates. While the POI ligand and E3 ligase ligand are usually constrained by binding requirements, the linker can be extensively modified (Table 1) and therefore becomes the main handle to tune overall molecular behavior.

**TABLE 1:** Examples of linker classes.

Linker class	Typical motifs/examples	Key properties
Alkyl	C3–C10 methylene chains	Highly flexible, lipophilic
PEG	PEG2–PEG6 (─CH_2_─CH_2_─O─)_*n*_	Flexible, polar, water‐soluble
Ether/amine heterochains	─O─, ─NH─, ─NR─ insertions	Tunable polarity and H‐bonding
Amide‐containing	─CONH─, ─NHCO─	More rigid, polar
Alkyne	─C≡C─	Linear, rigid
Triazole	1,4‐ or 1,5‐disubstituted 1,2,3‐triazoles	Rigid, polar, aromatic
Piperazine	1,4‐Disubstituted piperazines	Semirigid
Piperidine/saturated heterocycles	1,4‐Piperidines and related heterocycles	Semirigid
Cycloalkyl	1,3‐ or 1,4‐cyclohexylene and bridged bicycloalkyl units	Rigid, lipophilic
Aryl	1,3‐ or 1,4‐phenylene and related arylene units	Rigid, planar
Spiro	Disubstituted spirocyclic units, e.g., spiro[3.3]heptane	Highly rigid, 3D

Notably, within a matched PROTAC series (same warhead, same E3 ligand, same attachment points), linker modifications can affect degradation, physicochemical, and in vitro ADME properties (e.g., solubility and permeability) through partially independent mechanisms. As a result, the “optimal” linker for degradation is not necessarily the best one to ensure sufficient solubility, permeability, and overall developability.

Often referred to as “linkerology,” the trial‐and‐error optimization of linker regions in bifunctional molecules remains the most common strategy in PROTAC design. However, recent studies have highlighted the limitations of this largely empirical methodology and underscore the need for more rational linker design strategies that leverage and systematically exploit the growing body of available experimental data to accelerate the discovery of optimized PROTACs. To support rational linker design, the “property budget” has recently emerged [8]. This perspective considers the warhead, E3 recruiter, and linker as interdependent contributors to the overall physicochemical profile of the degrader, implying that linker properties cannot be interpreted in isolation. Moreover, what clearly emerges from the literature is the rapidly expanding number of synthesized PROTACs, alongside the increasing amount of associated experimental data [9]. Consequently, there is a growing need for simple computational strategies able to systematically organize the knowledge generated from previous PROTAC studies and support the design of subsequent synthetic iterations.

To address this need, we retrospectively investigated whether straightforward 2D linker descriptors could provide informative insights into the experimental behavior of PROTACs within matched series. Particular attention was given to their relationship with degradation efficiency and key physicochemical (lipophilicity) and in vitro ADME properties (solubility, permeability, and metabolic stability). In this regard, we implemented a multistep workflow. First, a systematic literature analysis was performed to identify suitable matched PROTAC series in which linker variation represented the main structural modification. Four representative datasets, including both CRBN and VHL derivatives fulfilling these criteria were selected (Table 2). These datasets contain multiple orthogonal experimental endpoints (e.g., degradation combined with permeability or physicochemical measurements). In the second step, linker substructures were identified using Bellerophon (https://bellerophon‐protac‐decomposing‐tool.streamlit.app/), a decomposition tool recently developed in our laboratory for the systematic fragmentation of datasets of PROTAC into warhead, linker, and E3 ligand [14]. Subsequently, a set of simple 2D descriptors (see below) were calculated to characterize linker properties, including parameters associated with hydrophobicity, polarity, flexibility, and molecular shape/topology. Finally, multivariate statistical analysis by principal component analysis (PCA) was employed to investigate potential correlations between linker descriptors and PROTACs experimental properties. Methodological aspects and limitations of this approach are discussed in more detail in the dedicated section below.

**TABLE 2:** PROTAC datasets considered in this study.

**POI** ^**a**^	**E3 ligase** ^**b**^	Property	Descriptor	***N*** ^**c**^	References
ERK5	VHL	Permeability, solubility	LogPe, concentration, µM	9	[10]
USP7	VHL	Degradation, solubility, drug–membrane interactions, lipophilicity	%Target degradation, logS, CHI_IAM_, elogD	7	[11]
IRAK4	CRBN	Degradation, permeability, ER, microsomal stability	DC_50_, Papp, ER, Clint, mL/min/mg	6	[12]
BRD4	CRBN	Degradation, solubility, PPB, lipophilicity	%Target degradation, logS, PPB %, elogD	10	[13]

Abbreviations: ER, efflux ratio; POI, protein of interest; PPB, plasma protein binding.

a PROTAC target.

b E3 ligase recruited by PROTAC molecules.

c Number of PROTACs discussed in this manuscript.

Overall, this article investigates whether 2D linker descriptors can be used as effective very early‐stage in silico tools in PROTAC drug discovery, specifically during lead identification, thereby helping to reduce the trial‐and‐error approaches that still largely characterize current PROTAC discovery workflows.

## 2 Results and Discussion

### 2.1 Methodological Aspects

The main objective was to determine whether principal component analysis (PCA), based on the five linker descriptors described below, could identify regions of chemical space associated with PROTACs displaying similar degradation efficiency, in vitro ADME behavior, and physicochemical profiles. To address this question, we developed a four‐step workflow, schematically illustrated in Figure 1. The outcome of the workflow is represented by a PCA biplot of the first two principal components (PCs), which provides a simultaneous visualization of both scores and loadings. In this representation, the scores describe the distribution of the linkers within the reduced chemical space, where nearby linkers share similar descriptor profiles, whereas the loadings indicate the contribution and correlation of the original variables to the PCs, with variables pointing in similar directions being positively correlated. To facilitate interpretation, each linker was additionally colored according to the experimental property measured for the corresponding PROTAC (e.g., red = worse profile and green = better profile).

**FIGURE 1:**
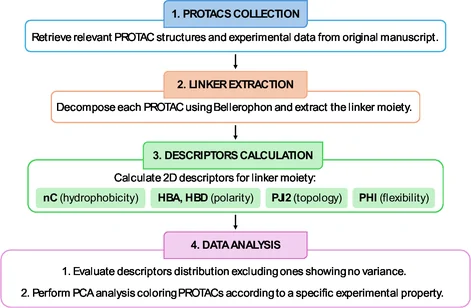
Flowchart describing the workflow presented in this article. First, relevant PROTAC structures and data were manually retrieved from the original papers. The second step was focused on extracting the linker structure from the SMILES of the chosen PROTACs with Bellerophon [14]. Then we calculated five descriptors to characterize the linker properties: nC (carbon atom number) for hydrophobicity, HBA (hydrogen bond acceptor count), HBD (hydrogen bond donor count) for polarity, PHI (Kier’s flexibility index) for flexibility and PJI2 (Petitjean shape index) as a measure of topological eccentricity and shape. Finally, after evaluating the distribution of the selected 2D descriptors and excluding those showing no variance within the series (i.e., descriptors with fewer than two distinct values), we performed a principal component analysis (PCA) to analyze the dataset.

A few comments associated with the proposed workflow are needed. First, only a limited number of published studies were appropriate for this purpose. In many cases, fewer than five linker‐matched PROTACs were reported, thereby limiting statistical significance. Ideally, the selected case studies would report both degradation data and one or more molecular properties. However, this was not always possible because, as previously noted by some of us, there is currently no standardized experimental pipeline for PROTAC evaluation, and physicochemical, solubility, and permeability data are often unavailable [15].

Another aspect that needs to be discussed concerns the definition of threshold values used to classify PROTACs into two categories for each investigated property (Tables S1–S8). Because of the heterogeneity among datasets, these thresholds were arbitrarily established individually for each series with the objective of maintaining a reasonably balanced distribution of compounds across the two classes.

A third consideration involves the use of Bellerophon for PROTAC decomposition. As highlighted in its original publication, the tool can generate multiple chemically valid decomposition patterns for a single PROTAC depending on how the boundaries between the linker and the protein ligands are assigned; consequently, the user must manually identify and select the correct solution. Additionally, users must always verify that the POI ligand is present within the tool’s reference dataset to avoid decomposition mismatches or errors (see case study 1 and 2).

The final comment relates to the selection of descriptors used to map the linker’s chemical space. Building upon our previous studies, which focused primarily on structural descriptors (e.g., molecular weight, MW) rather than descriptors property‐predictive descriptors (e.g., log *P*), we have introduced a few updates [16]. Specifically, we selected carbon atom count (nC) as an indicator of hydrophobicity; hydrogen bond donor (HBD) and acceptor (HBA) count as polarity descriptors; Kier’s flexibility index (PHI) to capture flexibility freedom; and the Petitjean Shape Index (PJI2) to quantify topological eccentricity. For the latter, values approaching 0 indicate compact or spherical architectures, whereas values nearing 1 denote highly elongated structures. Rather than reflecting absolute molecular size, PJI2 specifically captures the degree of spatial extension induced by different linker designs.

### 2.2 Case Studies

#### 2.2.1 Case 1. VHL‐Based PROTACs Targeting ERK5: Permeability and Solubility

Kihlberg et al. previously reported a series of nine VHL‐based ERK5 PROTACs featuring chemically diverse linkers (see Figure 2A and Tables S1 and S2). This structurally matched series was designed to systematically evaluate passive cell permeability as a prerequisite for establishing oral bioavailability. Employing a parallel artificial membrane permeability assay (PAMPA), the authors measured log *P*_e_ (cm/s) values, which revealed a remarkably wide dynamic range spanning nearly three orders of magnitude. Notably solubility data for most PROTACs were also reported.

**FIGURE 2:**
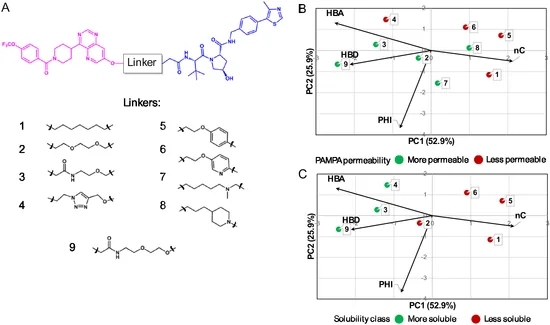
(A) VHL‐PROTAC structures are shown. PC1 versus PC2 of linkers 2D descriptors HBA, HBD, nC, and PHI; (B) PROTACs are colored according to PAMPA permeability classification (green permeable, red less permeable); (C) PROTACs are colored according to solubility classification (green more soluble, red less soluble).

PROTAC structures were first submitted to Bellerophon to identify their individual components. Since this initial attempt triggered a “linker inconsistency error,” we verified that the warhead was not present in the default library [14]. After manually adding the warhead to the library, the dataset was successfully decomposed, yielding a single solution for each PROTAC. The chemical space of this warhead series relative to the default Bellerophon library is illustrated in Figure S1A, while the resulting linker structures are shown in Figure 2A.

Two‐dimensional descriptors were calculated for the nine linkers and used to construct the PCA model. Notably, PJI2 was excluded because it had zero variance. The first two PCs accounted for approximately 78% of the total variance. In the corresponding PCA biplot, linkers are represented as filled circles and colored according to the permeability value associated with the corresponding PROTACs (e.g., red = lower values and green = higher values; Figure 2B).

Notably, poorly permeable PROTACs were well‐separated from the more permeable compounds within the PCA space. The analysis also suggested that increasing flexibility (PHI) is expected to improve permeability. Solubility data was also available for six of the nine compounds (excluding compounds 7 and 8). The more soluble PROTACs clustered in the region are characterized by higher polarity and lower nC values (HBA and HBD, Figure 2C). In the PCA space, this soluble region mostly overlaps with the permeable region (Figure 2C). For instance, within this overlapping zone, compounds 3 and 9 exhibit both high solubility and high permeability. Conversely, compound 4 serves as an outlier to this trend, demonstrating high polarity but poor permeability.

#### 2.2.2 Case 2. VHL‐Based PROTACs Targeting USP7: Degradation, Solubility, and Lipophilicity

This study describes the rational optimization of VHL‐based USP7 degraders with the aim of improving their developability (Table 2). A series of 25 PROTACs were reported in the article; however, only seven compounds share the same warhead and E3 ligase ligand, enabling a direct assessment of linker effects. The structures relevant to our discussion are shown in Figure 3A. Linker modifications span from a homologous series of linear alkyl chains of different lengths to more polar architectures or cyclic nitrogen‐ and amide‐containing motifs. For these PROTACs degradation, solubility, and lipophilicity (elogD, a chromatographic method that determines lipophilicity in octanol/water and CHI_IAM_ that is an estimate for drug‐membrane phospholipids interaction) data have been measured (polarity expressed as EPSA was not considered because of the limited variability within the series).

**FIGURE 3:**
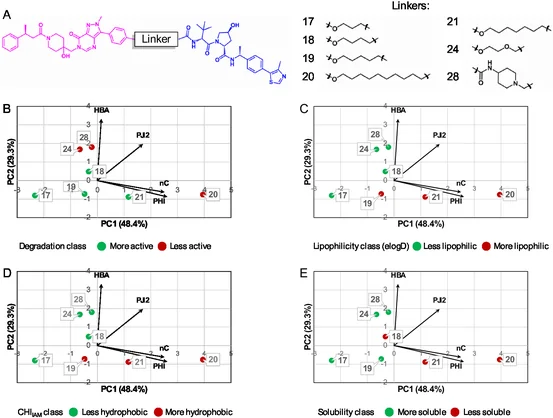
(A) PROTAC series warhead, E3 ligand and linkers are shown. The other panels show PC1 versus PC2 of linkers 2D descriptors HBA, PJI2, nC and PHI; (B) PROTACs are colored according to degradation classification (green active, red inactive); (C) compounds are colored according to lipophilicity classification (elogD; green less lipophilic, red more lipophilic); (D) compounds are colored according to CHI_IAM_ hydrophobicity classification (green less hydrophobic, red more hydrophobic); and (E) compounds are colored according to solubility classification (green more soluble, red less soluble).

During the initial PROTAC decomposition, Bellerophon triggered a “linker inconsistency” error due to the absence of the warhead from the tool’s default library (Figure S1). The manual inclusion of this warhead in the library resolved the issue, enabling successful decomposition. In instances where the tool generated multiple decomposition patterns, we strictly selected the single solution that aligned with the structural definitions outlined in the original publication (all alternative solutions are provided in the Supporting Information). Following the calculation of 2D descriptors for all degraders, PCA was performed. Notably, the HBD descriptor was excluded from this step as it exhibited zero variance (retaining a constant value of 1) across the entire dataset (Table S4).

The resulting PCA model captures 78% of the total dataset variance, with 48.4% explained by PC1 and 29.3% by PC2. Overlaying the experimental data revealed a clear spatial separation between PROTACs with the highest and lowest degradation efficiencies (Figure 3B), a trend driven primarily by higher PJI2 and HBA values. Similarly, the lipophilicity profiles (elogD and CHI_IAM_) successfully partitioned the chemical space between well‐ and poorly lipophilic compounds (Figure 3C,D). However, these parameters were governed by a different set of descriptors; specifically, higher nC and PHI values strongly correlated with increased lipophilicity. In contrast, a less discernable trend could be identified for aqueous solubility (Figure 3E).

#### 2.2.3 Case 3. CRBN‐Based PROTACs Targeting IRAK4: Degradation, Permeability, and Microsomal Stability

This study describes the discovery and optimization of KT‐474 (compound 17 in the article), a potent, selective, and orally bioavailable IRAK4 degrader developed for autoimmune diseases. The authors systematically optimized the IRAK4 binder, linker composition, and overall physicochemical properties to balance degradation potency, permeability, metabolic stability, and oral bioavailability. To rigorously isolate the effect of linker composition within a consistent chemical framework, several PROTACs were excluded from our analysis. The six degraders retained for the analysis are shown in Figure 4A. For these PROTACs degradation, permeability (passive and efflux ratio, ER), and human microsomal stability were reported.

**FIGURE 4:**
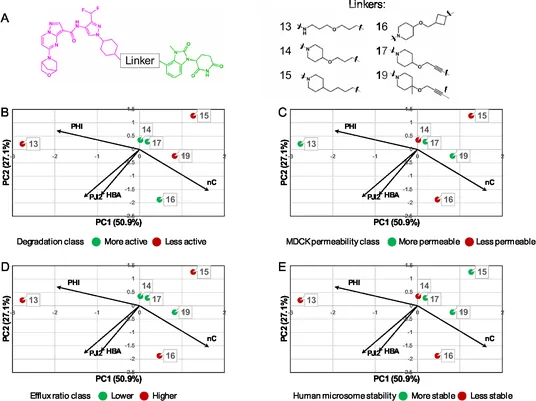
(A) IRAK4 PROTAC series warhead, E3 ligand, and linkers are shown. The other panels show PC1 versus PC2 of linkers 2D descriptors HBA, PJI2, nC, and PHI; (B) PROTACs are colored according to degradation classification (green active, red inactive); (C) compounds are colored according to MDCK permeability classification (green more permeable, red less permeable); (D) compounds are colored according to efflux ratio classification (green lower, red higher); and (E) compounds are colored according to human microsomal metabolic stability classification (green more stable, red less stable).

Because the IRAK4 warhead was already present in the default library, Bellerophon seamlessly decomposed this PROTAC series into its corresponding components. Following 2D descriptor calculation, HBD count was omitted from the multivariate analysis because it remained constant across all selected linkers (Table S6). The resulting two‐dimensional PCA biplot accounted for 78% of the total structural variance, with PC1 and PC2 explaining 50.9% and 27.1%, respectively. To map this chemical space to in vitro performance, experimental values for degradation activity, MDCK cellular permeability, MDR1 efflux ratios, and human liver microsomal stability were overlaid onto the score plot. Unlike the previous case studies, no distinct partitioning between high‐ and low‐performing candidates could be identified for any of the evaluated endpoints. The absence of a clear pattern may be interpreted in the context of the property budget concept as the overall physicochemical profile of these PROTACs may be largely influenced by the IRAK4 warhead and CRBN ligand, thereby limiting the impact of the relatively subtle linker modifications. Consequently, variation in simple 2D linker descriptors alone may not be sufficient to produce clear differences in the investigated degradation and in vitro developability endpoints.

#### 2.2.4 Case 4. CRBN‐Based PROTACs Targeting BRD4: Degradation, Protein Binding, Solubility, and Lipophilicity

As a fourth representative case study, we selected the series of matched‐pair PROTACs reported by Steinebach and coworkers, who developed benzamide‐based cereblon recruiters as alternatives to classical immunomodulatory imide drug (IMiDs) ligands. In this series, the authors systematically varied linker composition and rigidity while maintaining the same BRD4 warhead and CRBN‐binding scaffold, enabling a direct evaluation of linker effects on degradation efficiency and physicochemical properties (protein binding, solubility, and lipophilicity expressed as elogD). The chemical structures of the 10 investigated degraders (43a–k) are shown in Figure 5A.

**FIGURE 5:**
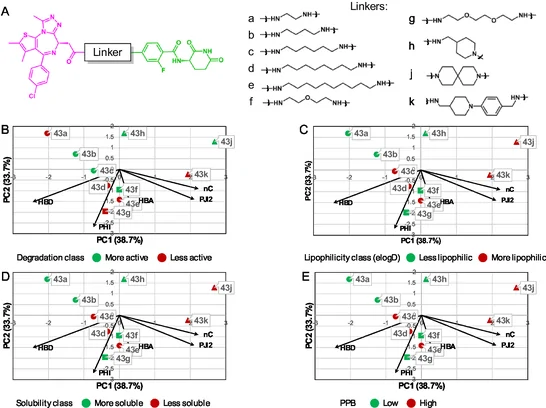
(A) PROTAC series warhead, E3 ligand and linkers are shown. (B–E) Biplots of PC1 versus PC2 calculated on the five 2D linker descriptors (HBA, HBD, PJI2, nC, and PHI). Compounds are distinguished by geometric shapes according to their linker topology: circles represent aliphatic chains, squares denote pegylated linkers, and triangles highlight cyclic architectures. (B) PROTACs are colored according to degradation classification (green active, red inactive); (C) compounds are colored according to lipophilicity classification (elogD; green less lipophilic, red more lipophilic); (D) compounds are colored according to solubility classification (green more soluble, red less soluble); and (E) compounds are colored according to plasma protein binding (PPB) classification (low in green, high in red).

Degraders were decomposed using Bellerophon, and several solutions for each PROTAC were produced (Supporting Information). After choosing the solution of interest, 2D descriptors were calculated, and the PCA analysis was performed. Unlike the previous cases, all five evaluated 2D descriptors (HBA, HBD, PJI2, nC, and PHI) exhibited nonzero variance across the linker series and were retained in the model. The resulting two‐dimensional biplots capture 72.4% of the total dataset variance, with PC1 accounting for 38.7% and PC2 explaining 33.7% (Figure 5B–E). Although mapping the experimental data failed to reveal a global, distinct separation between well‐ and poorly performing candidates across the entire dataset, localized trends emerged within the aliphatic subclass 43a‍–‍e. For these specific derivatives, clear linker‐dependent patterns were identified for lipophilicity, solubility, and protein binding: shorter linkers consistently correlated with lowered lipophilicity, improved solubility, and diminished plasma protein binding. In contrast, target degradation efficiency followed a distinct, nonlinear pattern that could not be readily rationalized within this simplified 2D space.

## 3 Conclusions

The PROTAC discovery pipeline spans from hit identification to lead optimization, where degradation efficiency and molecular properties must be optimized simultaneously. At early stages, simple computational and data‐driven approaches can support candidate prioritization and reduce reliance on purely empirical trial‐and‐error strategies.

In this study, we analyzed two CRBN‐based and two VHL‍‐‍based PROTAC series using a simple workflow combining the freely available decomposition tool Bellerophon with PCA based on selected 2D linker descriptors and experimental PROTAC data. Remarkably, without an automated decomposition strategy like the one provided by Bellerophon, linker extraction from published PROTAC structures would rely on manual annotation, which is time‐consuming, difficult to standardize across datasets, and prone to subjective interpretation, especially when linker boundaries overlap with ligand moieties. Bellerophon addresses this issue through a deterministic rule‐based workflow that ensures structural consistency and atom conservation during PROTAC splitting.

Importantly, the quantitative viability of this minimalist descriptor set is supported by the consistently high proportion of variance captured across all four series, with the first two principal components explaining 72%–78% of the total dataset variance. Despite the exploratory nature of the analysis and the paucity of matched‐pair series in the literature, this robust low‐dimensional projection revealed distinct E3 ligase‐dependent behaviors: 2D linker descriptors exerted a clearer, more predictable influence on degradation and molecular properties in VHL‐recruiting PROTACs than in CRBN‐based degraders, where the heavy property budget of the warhead and E3 ligase ligand dampened isolated linker effects. While the limited size of the examined datasets precludes broad generalizations, this work establishes a qualitative framework to guide very early‐stage PROTAC design. By rationalizing how specific linker features modulate degradation efficiency, ADME parameters, and physicochemical profiles, this workflow may effectively minimize redundant synthetic efforts. Crucially, applying this approach prospectively requires only a modest initial investment: a chemist can synthesize a baseline matrix of 5–6 structurally diverse linkers, map their experimental trends, and project new design ideas into the established PCA space to prioritize candidates falling within the optimized cluster quadrant. Ultimately, scaling this methodology to larger, proprietary industrial datasets represents a highly valuable next step, highlighting the vital role of academia–industry collaborations in refining predictive TPD tools.

It is important to note that, at more advanced stages of optimization, additional properties such as chameleonicity and conformational adaptability are expected to become critical for balancing permeability, solubility, metabolic stability, and oral bioavailability in these large beyond‐Rule‐of‐5 molecules. Indeed, the dynamic ability of PROTACs to modulate their exposed properties through conformational changes is increasingly recognized as a key determinant of successful pharmacokinetic behavior. In this context, different stages of drug discovery are likely to require distinct experimental and computational tools: while simple physicochemical descriptors and qualitative trends may be sufficient to guide very early design and prioritization, later optimization stages demand more sophisticated approaches capable of capturing three‐dimensional conformational behavior, intramolecular interactions, and environment‐dependent properties. This further highlights the need for integrated and stage‐appropriate methodologies to efficiently support the development of complex heterobifunctional degraders.

## 4 Methods

### 4.1 PROTAC Decomposition and Structural Curation

SMILES strings and original compound names were retrieved from the literature and submitted to Bellerophon (https://bellerophon‐protac‐decomposing‐tool.streamlit.app/) for structural decomposition [14]. Whenever a decomposition error occurred, the tool’s default libraries were manually inspected to check for the corresponding warhead and E3 ligand. Missing fragments were manually added to the library, and the decomposition was reexecuted. In cases where Bellerophon generated multiple structural solutions, the single solution that strictly matched the structural definitions outlined by the original authors was selected.

### 4.2 Descriptor Calculation and PCA

Linker properties were calculated using AlvaDesc (v2.0.6, Alvascience, www.alvascience.com). Principal component analysis (PCA) was performed using DataWarrior (v05.05.00, DataWarrior, https://openmolecules.org/datawarrior/) and refined graphically in Microsoft Excel (v2604, Microsoft 365,  https://www.microsoft.com/it‐it/microsoft‐365/excel). Prior to running the PCA, a variance filter was applied: any 2D descriptor showing zero variance (i.e., holding the exact same value across all compounds in a series) was excluded from the analysis.

## Funding

This study was supported by Università degli Studi di Torino (Grant Ricerca Locale ex‐60%, Bando2024).

## Conflicts of Interest

The authors declare no conflicts of interest.

## Supporting information

Supplementary Material

Supplementary Material

## Data Availability

The data that support the findings of this study are available on request from the corresponding author. The data are not publicly available due to privacy or ethical restrictions.
