# Supplementary material for: Limiting Trial‐and‐Error: Linker‐Based 2D Descriptor Analysis to Support Very Early Proteolysis‐Targeting Chimeras Design
Source: ChemMedChem. 2026 Oct 11;21(19):e70532. doi: 10.1002/cmdc.70532 (PMC13655484; doi:10.1002/cmdc.70532)
Supplement: Supplementary file 1 [file CMDC-21-e70532-s002.pdf]

Limiting trial-and-error: Linker-Based 2D Descriptor Analysis to Support Very Early PROTAC Design

Giulia Apprato [1], Serena Traversa [1], Giulia Caron *[1] and Giuseppe Ermondi *[1]

[1] Department of Molecular Biotechnology and Health Sciences
University of Turin
Piazza Nizza 44 bis
E-mail: giuseppe.ermondi@unito.it, giulia.caron@unito.it

Supporting Information

LIST OF TABLES

Table S1. Case study 1 dataset: experimental data……………………………………………………..2

Table S2. Case study 1 dataset: 2D descriptors……………………………………………………….2

Table S3. Case study 2 dataset: experimental data…………………………………………………..3

Table S4. Case study 2 dataset: 2D descriptors. ……………………………………………………..3

Table S5. Case study 3 dataset: experimental data. ……………………………………………....4

Table S6. Case study 3 dataset: 2D descriptors. ……………………………………………………..4

Table S7. Case study 4 dataset: experimental data.…………………………………………………5

Table S8. Case study 4 dataset: 2D descriptors. ……………………………………………………..5

LIST OF FIGURES

Figure S1. Chemical space analysis of manually appended warheads…...……………………………6

Table S1. Case study 1 dataset: experimental data. Thresholds: PAMPA permeability (log Pe) ≥ -7; Solubility threshold ≥ 5 µM.

Name	PAMPA (logPe)	PAMPA class (-7)	Solubility (µM)	Solubility class (5 µM)
**c1**	-8.29	low	0.1	low
**c2**	-5.42	high	0.64	low
**c3**	-6.21	high	17	high
**c4**	-7.94	low	12.1	high
**c5**	-7.56	low	0.36	low
**c6**	-7.28	low	0.52	low
**c7**	-6.67	high	-	-
**c8**	-6.9	high	-	-
**c9**	-6.3	high	6.78	high

Table S2. Case study 1 dataset: 2D descriptors.

Name	Linker SMILES	nC	PHI	HBD	HBA	PJI2
**c1**	CCCCCCC	7	6	0	0	1
**c2**	CCOCCOC	5	5.856	0	2	1
**c3**	COCCNC(C)=O	5	4.51	1	3	1
**c4**	CCn1cc(CO)nn1	5	1.926	1	3	1
**c5**	CCOc1ccccc1	8	2.28	0	1	1
**c6**	CCOc1cccnc1	7	2.221	0	2	1
**c7**	CCCCCCNC	7	6.927	1	1	0.75
**c8**	CCCC1CCNCC1	8	3.055	1	1	1
**c9**	CC(=O)NCCOCCO	6	6.381	2	4	1

Table S3. Case study 2 dataset: experimental data. Thresholds: Degradation ≥ 75%; CHI_{IAM} ≤ 36; Solubility (logS) ≥ -7.1; Lipophilicity (elogD) ≤ 2.8.

Name	Degradation	Degradation class (75%)	CHI_{IAM}	CHI_{IAM} class (36)	logS	logS class (-7.1)	elogD	elogD class (2.8)
**17**	92	active	34.6	low	-6.27	high	2.6	low
**18**	96	active	35.6	low	-7.34	low	2.7	low
**19**	92	active	36.5	high	-6.98	high	2.9	high
**20**	48	inactive	45.5	high	-7.77	low	4.1	high
**21**	92	active	39.6	high	-7.52	low	3.3	high
**24**	71	inactive	34.5	low	-6.06	high	2.5	low
**28**	23	inactive	33.3	low	-5.83	high	2.2	low

Table S4. Case study 2 dataset: 2D descriptors.

Name	Linker SMILES	nC	PHI	HBD	HBA	PJI2
**17**	CCCO	3	2.932	1	1	0.5
**18**	CCCCO	4	3.93	1	1	1
**19**	CCCCCO	5	4.929	1	1	0.667
**20**	CCCCCCCCCCO	10	9.926	1	1	1
**21**	CCCCCCCO	7	6.927	1	1	0.75
**24**	COCCO	3	3.861	1	2	1
**28**	CN1CCC(NC=O)CC1	7	2.842	1	3	0.75

Table S5. Case study 3 dataset: experimental data. Thresholds: Degradation (DC_{50}) ≤ 8 nM; MDCK permeability ≥ 7x10^{-6} cm/s; Efflux ratio (ER) ≤ 8; Human liver microsomal intrinsic clearance ≤ 80 mL/min/kg.

Name	Degradation (DC_{50} nM)	Degradation class (8 nM)	Permeability (7x10^{-6} cm/s)	Permeability Class (7)	ER	ER class (8)	Microsomal Clint (mL/minkg)	Microsomal Clint Class (80)
**13**	9	inactive	16.2	permeable	14.2	high	268	unstable
**14**	7	active	6.42	not permeable	7.95	low	111	unstable
**15**	39	inactive	4.46	not permeable	9.54	high	54.4	stable
**16**	5	active	3.86	not permeable	9.54	high	82.9	unstable
**17**	2	active	8.07	permeable	2.83	low	32.7	stable
**19**	17	inactive	10	permeable	2.01	low	76.8	stable

Table S6. Case study 3 dataset: 2D descriptors.

Name	Linker_SMILES	nC	PHI	HBD	HBA	PJI2
**13**	CCCOCCCNC	7	7.853	1	2	1
**14**	CCCOC1CCNCC1	8	3.766	1	2	0.75
**15**	CCCCC1CCNCC1	9	3.811	1	1	0.75
**16**	C1CC(COC2CCNCC2)C1	10	3.01	1	2	1
**17**	C#CCOC1CCNCC1	8	3.277	1	2	0.75
**19**	C#CCOC1(C)CCNCC1	9	2.925	1	2	0.75

Table S7. Case study 4 dataset: experimental data. Thresholds: Degradation ≥75%; Lipophilicity (elogD) ≥ 2.7; Plasma protein binding (PPB) ≥ 94%; Solubility (logS) ≥ -2. *ND values: solubility lower than the detection limit (<1 μg/mL).

Name	Degradation (%)	Degradation class (75)	Lipophilicity (elogD)	Lipophilicity class (2.7)	PPB %	PPB class (94)	Solubility (logS)*	Solubility class (-2)
**43a**	70	inactive	2.1	low	91	low	-4.8	high
**43b**	85	active	2.3	low	92	low	-4.5	high
**43c**	80	active	2.7	high	94	high	ND	low
**43d**	69	inactive	3.3	high	95	high	ND	low
**43e**	65	inactive	3.8	high	96	high	ND	low
**43f**	81	active	2.2	low	90	low	-4.3	high
**43g**	60	inactive	2.3	low	90	low	-3.9	high
**43h**	91	active	2.5	low	93	low	-5.8	high
**43j**	83	active	3.1	high	95	high	ND	low
**43k**	37	inactive	2.9	high	95	high	ND	low

Table S8. Case study 4 dataset: 2D descriptors.

Name	Linker SMILES	nC	PHI	HBD	HBA	PJI2
**43a**	NCCN	2	2.865	4	2	0.5
**43b**	NCCCCN	4	4.858	4	2	0.667
**43c**	NCCCCCCN	6	6.855	4	2	0.75
**43d**	NCCCCCCCCN	8	8.853	4	2	0.8
**43e**	NCCCCCCCCCCN	10	10.851	4	2	0.833
**43f**	NCCOCCN	4	5.785	4	3	1
**43g**	NCCOCCOCCN	6	8.706	4	4	0.8
**43h**	NCC1CCNCC1	6	2.306	3	2	0.667
**43j**	C1CC2(CCN1)CCNCC2	9	2.143	2	2	1
**43k**	CNc1ccc(N2CCC(CN)CC2)cc1	13	3.937	3	3	0.833

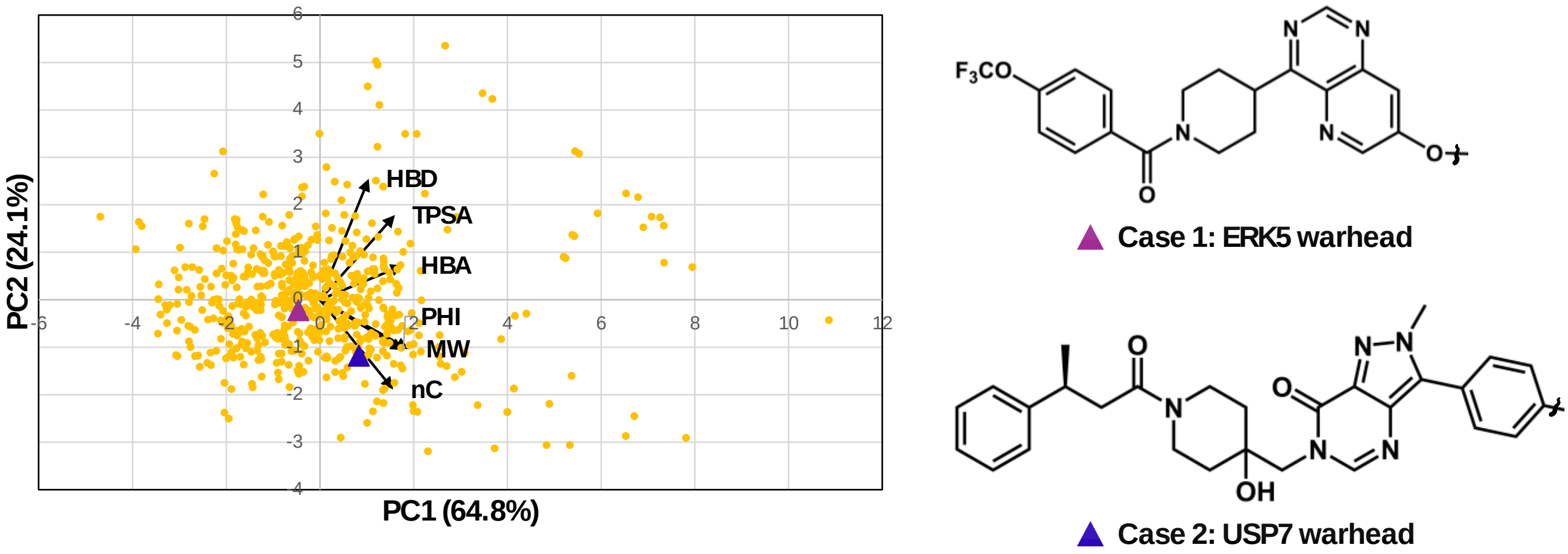

Figure S1. Chemical space analysis of manually appended warheads. Principal Component Analysis (PCA) projection of the target ERK5 (Case Study 1) and USP7 (Case Study 2) warhead structures within the chemical space defined by the default Bellerophon library[10].
